# Uncovering the mutation-fixation correlation in short lineages

**DOI:** 10.1186/1471-2148-7-168

**Published:** 2007-09-21

**Authors:** Eric J Vallender, Bruce T Lahn

**Affiliations:** 1 Howard Hughes Medical Institute, Department of Human Genetics, and Committee on Genetics, University of Chicago, USA; 2 New England Primate Research Center, Harvard Medical School, USA

## Abstract

**Background:**

We recently reported a highly unexpected positive correlation between the fixation probability of nonsynonymous mutations (estimated by ω) and neutral mutation rate (estimated by *K*_s_) in mammalian lineages. However, this positive correlation was observed for lineages with relatively long divergence time such as the human-mouse lineage, and was not found for very short lineages such as the human-chimpanzee lineage. It was previously unclear how to interpret this discrepancy. It may indicate that the positive correlation between ω and *K*_s _in long lineages is a false finding. Alternatively, it may reflect a biologically meaningful difference between various lineages. Finally, the lack of positive correlation in short lineages may be the result of methodological artifacts.

**Results:**

Here we show that a strong positive correlation can indeed be seen in short lineages when a method was introduced to correct for the inherently high levels of stochastic noise in the use of *K*_s _as an estimator of neutral mutation rate. Thus, the previously noted lack of positive correlation between ω and *K*_s _in short lineages is due to stochastic noise in *K*_s _that makes it a far less reliable estimator of neutral mutation rate in short lineages as compared to long lineages.

**Conclusion:**

A positive correlation between ω and *K*_s _can be observed in all mammalian lineages for which large amounts of sequence data are available, including very short lineages. It confirms the authenticity of this highly unexpected correlation, and argues that the correction likely applies broadly across all mammals and perhaps even non-mammalian species.

## Background

Point mutations in coding regions of genes can be classified into two categories: synonymous and nonsynonymous. In mammals, synonymous mutations are largely neutral, though they may sometimes experience weak selection [1]. Nonsynonymous mutations, in contrast, are frequently subject to strong selection. The rate of fixed synonymous (or nonsynonymous) substitutions is often denoted as *K*_s _(or *K*_a_). *K*_s _is typically treated as a reasonable proxy for neutral mutation rate, and the *K*_a_/*K*_s _ratio (abbreviated ω) is often used as a measure for the fixation probability of nonsynonymous mutations scaled to neutral expectation.

Recently, we discovered a strong positive correlation between ω and *K*_s _in several mammalian lineages, arguing that the fixation probability of nonsynonymous mutations in a gene is positively corrected with the gene's neutral mutation rate [2]. This finding is highly unexpected under the classical neutral theory of molecular evolution, which argues that the fixation probability of nonsynonymous mutations is determined by (and serves as an estimator for) selective pressure, and as such, should be independent of neutral mutation rate [3-5]. Conventionally, therefore, ω should not show any positive correlation with *K*_s_, and in fact, simulations demonstrate that only an inverse correlation should in theory exist between ω and *K*_s _due to a mathematical artifact [2] (see below). The observation of a strong positive correction between ω and *K*_s _therefore challenges the current paradigm of molecular evolution, and necessitates a reexamination of the widely held assumption that ω is determined primarily by selective pressure independent of neutral mutation rate.

There is, however, an important caveat to this finding. The positive correlation between ω and *K*_s _was observed in mammalian lineages with relatively large sequence divergence, specifically, the human-mouse, human-rabbit, and mouse-rat lineages [2] (the average *K*_s _of these lineages being 0.48, 0.38 and 0.18, respectively), but not found in the human-chimpanzee or the human-macaque lineages, which have much lower levels of sequence divergence (the average *K*_s _being 0.012 and 0.063, respectively). Instead, an inverse correlation between ω and *K*_s _was seen in these latter short lineages [2]. This is troubling because it leaves open the possibility that the positive correlation between ω and *K*_s _observed in long lineages may actually be an artifact associated with the imprecise calculation of *K*_a _or *K*_s_. In particular, multiple hits at any given nucleotide position can occur with high probability in long lineages. Such multiple hits, difficult to correct since only one or zero changes can be observed at a given nucleotide position, can constitute a major source of error in the calculation of *K*_a _and, even more so, *K*_s_. It is formally possible, therefore, that errors stemming from the improper correction of multiple hits along with some other unknown factors have systematically biased the calculation of *K*_a _and *K*_s _in long lineages, in a manner that creates an artifactual positive correlation between ω and *K*_s_.

We have argued previously that this inverse correlation may be due to the effect of stochastic noise on *K*_s_, which affects short lineages more profoundly than long lineages. Specifically, stochastic deviation of *K*_s _from the true underlying neutral mutation rate (μ) represents a much greater fraction of μ in short lineages as compared to long lineages, and our simulations showed that this could indeed lead to a strong inverse correlation between ω and *K*_s _in very short lineages such as the human-chimpanzee lineage because *K*_s _is also used as the denominator in the calculation of ω (which equals *K*_a_/*K*_s_) (see Supplementary Material in [2]).

If the failure to observe a positive correlation between ω and *K*_s _in short lineages is indeed due to the inherently high levels of stochastic noise affecting *K*_s _in short lineages, then a reasonable correction of noise in *K*_s _might be able to bring out the positive correlation again. Here, we show that by introducing a method that corrects for stochastic noise in *K*_s_, a strong positive correlation between ω and *K*_s _indeed becomes observable in short lineages. This result strengthens the authenticity of this highly unexpected correlation, and argues that a biological mechanism (rather than a methodological artifact) is relating the fixation probability of nonsynonymous mutations to mutation rate.

## Results

We generated a set of 6,779 genes each with orthologs from five primate and rodent species for which large-scale genome sequences are currently available: human, chimpanzee, rhesus macaque, mouse, and rat. A subset of 5,831 genes also included orthologs from dog. To ensure that the current data set was consistent with our earlier results based on a separately derived data set [2], we sought to recapitulate the positive correlation between ω and *K*_s _in human-mouse and mouse-rat comparisons. Indeed, for both lineages, a highly robust correlation exists and binning of genes creates a visually striking representation of the correlation (Additional file 1). We also plotted human-dog and mouse-dog values and observed correlations similar to that seen in the human-mouse lineage (data not shown). To ensure that the correlation is not restricted to genes with orthologs in all the species sampled, we also obtained data sets containing only pairwise orthologs. Again, we observed correlations that are essentially the same as that seen in the five- or six-way ortholog sets (data not shown).

We then considered the human-chimpanzee and human-macaque ortholog pairs within the complete data set. As expected for these short lineages, plotting ω against *K*_s _showed that not only is there not a positive correlation, but there is a strong inverse correlation between these two parameters for the human-chimpanzee lineage (Additional file 2). We have speculated previously that this inverse correlation is due to the inherently high levels of stochastic variation in *K*_s _when it is used as a proxy for neutral mutation rate in short lineages [2]. If this is correct, then the strength of the inverse correlation should be stronger in the human-chimpanzee lineage than in the human-macaque lineage because the former is shorter and therefore suffers from an even higher level of stochastic noise in *K*_s_. This is precisely what we observed (Additional file 2). Indeed, the general finding is that as evolutionary distance of a lineage decreases (and hence the stochastic noise associated with *K*_s _relative to neutral mutation rate increases), the relationship between ω and *K*_s _goes from a strong positive correction in the case of long lineages progressively toward a strong inverse correlation in the case of very short lineages.

In molecular evolutionary studies, *K*_s _is frequently used as a proxy for neutral mutation rate (μ). It is often forgotten, however, that mutational events are a discreet process and as such are subject to stochastic variation. Over short periods of evolutionary time, this stochastic variation is often large relative to the true underlying neutral mutation rate. As evolutionary time lengthens and the number of mutational events increases, stochastic variation decreases relative to neutral mutation rate. This is clearly demonstrated by computer simulations in our previous study [2].

High levels of stochastic noise in *K*_s _can lead to an artifactual inverse correlation is because *K*_s _is in both parameters being corrected, and it is the denominator of ω. Mathematically, by just correcting for noise on the x-axis (*K*_s_), the artifactual inverse correlation should go away even if there is still considerable noise on the y-axis. This prediction was clearly borne out by our previous simulation studies [2]. We therefore decided to focus on devising a means to correct for noise in *K*_s _only, and to examine if such correction can eliminate the artifactual inverse correlation between ω and *K*_s _in short lineages. This may reveal the underlying positive correlation, if any, that has been obscured.

**Figure 1:**
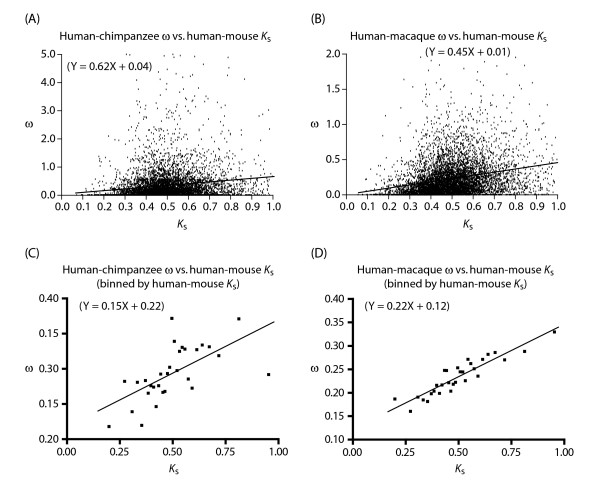
Positive correlation between ω of the short human-chimpanzee or human-macaque lineage and *K*_s _of the long human-mouse lineage. In cases where genes are binned, all the genes used in the analysis are divided into bins based on ascending *K*_s _(*i.e*., the first bin contains genes with the lowest *K*_s _values and the last bin contains genes with the highest *K*_s _values, *etc*.). There are 200 genes per bin except for the last bin, which contains whatever number of remaining genes that is 200 or less.

One way to accomplish this is to use the *K*_s _value from orthologs of the same gene but in a longer lineage (*i.e*., *K*_s _between a pair of species with greater divergence time). Indeed, when we plotted ω of the human-chimpanzee lineage against *K*_s _of the corresponding human-mouse lineage, not only did the inverse correlation disappear, but a positive correlation as typically observed in long lineages was seen (Figure 1). The same result should, and did, occur when human-macaque ω was plotted against human-mouse *K*_s _(Figure 1). We note that an implicit assumption in this approach is that the neutral mutation rate of a gene is correlated across different mammalian lineages [6-9]. Although the existence of such a correlation has been contentious [6,7,10], we were able to verify it in our data set independent of GC content, including not only *K*_s _but also *K*_4 _(Additional file 3).

**Figure 2:**
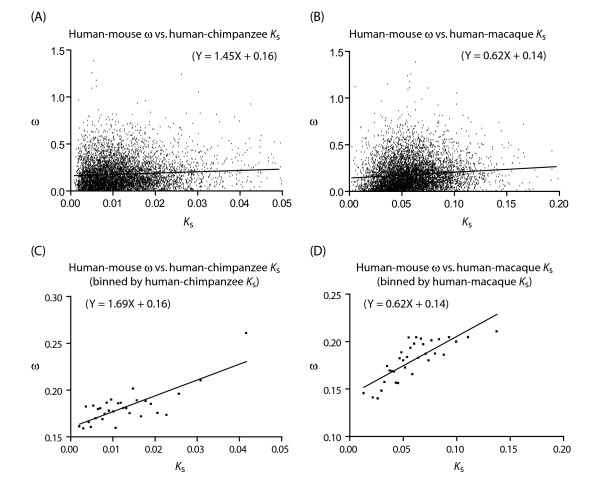
Positive correlation between ω of the long human-mouse lineage and *K*_s _of the short human-chimpanzee or human-macaque lineage. The binning of genes follows the convention in Figure 1.

In a similar manner, it is possible to correct for variation in ω apart from *K*_s_. By using the *K*_s _value from the human-chimpanzee lineage but ω from human-mouse, we were also able to observe the positive correlation (Figure 2). It is interesting to note the differences between these two approaches. When *K*_s _is corrected, the positive correlation appears more robust than when ω is corrected. This is likely due to a the fact that *K*_s _is used as the denominator in the calculation of ω.

**Figure 3:**
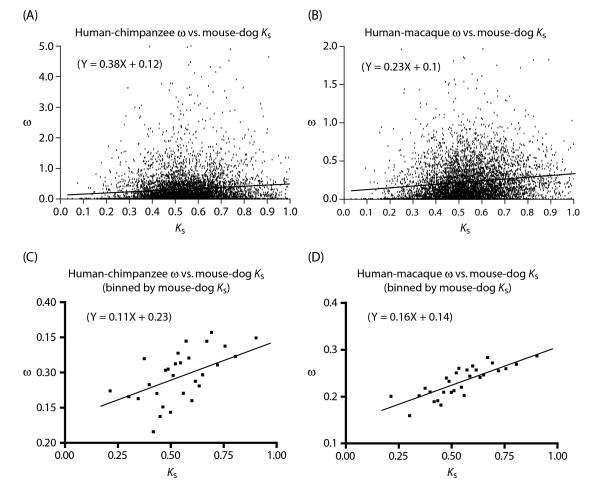
Positive correlation between ω of the short human-chimpanzee or human-macaque lineage and *K*_s _of the long mouse-dog lineage. The binning of genes follows the convention in Figure 1.

In the above analyses, the lineage used to calculate ω is entirely subsumed by the lineage used to calculate *K*_s _(*e.g*., when human-chimp ω was plotted against human-mouse *K*_s_). This may introduce confounding effects. We therefore also plotted human-chimp or human-macaque ω against mouse-dog *K*_s_. We found that a rather similar positive correlation between ω and *K*_s _exists despite a complete lack of shared descent between the lineage used to calculate ω and the lineage used to calculate *K*_s _(Figure 3)

**Figure 4:**
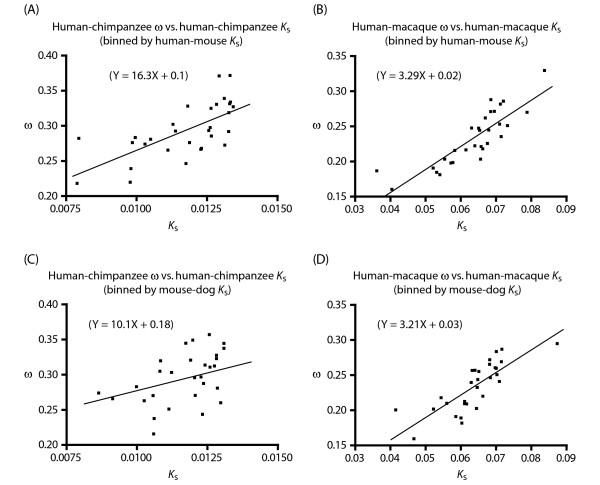
Positive correlation between bin-average ω of a short lineage and bin-average *K*_s _of that same short lineage when genes are binned by *K*_s _of a long lineage. The binning of genes follows the convention in Figure 1.

In the initial analysis, ω of a short lineage is plotted against *K*_s _of a long lineage. This reveals a positive correction presumably because *K*_s _of the long lineage is a more accurate estimator of neutral mutation rate than *K*_s _of the short lineage even for the short lineage. The ideal situation, however, is to plot ω of a short lineage against *K*_s _of the same short lineage, and do so in a manner that corrects for the stochastic noise in *K*_s_. One approach to correct for the noise in *K*_s _affecting individual genes is to bin genes and plot the bin-average ω against bin-average *K*_s_. Given that *K*_s _of the long lineage is a more accurate proxy for neutral mutation rate, we binned genes based on *K*_s _in the long lineage even though bin-average ω and *K*_s _values were all derived from the short lineage. Using this approach, we first plotted bin-average ω against bin-average *K*_s _in the human-chimpanzee lineage, using human-mouse *K*_s _to bin genes. This revealed a robust positive correlation between ω and *K*_s _(Figure 4). A similar positive correlation was seen between bin-average ω and *K*_s _in the human-macaque lineage when human-mouse *K*_s _was again used to bin genes (Figure 4). Comparable results were also obtained for either the human-chimpanzee or human-macaque lineage when mouse-dog *K*_s _was used to bin genes.

We note that the accuracy of *K*_a _and *K*_s _can be compromised by polymorphisms and that the effect is stronger for short lineages. This is because in short lineages, a considerable fraction of observed sequence differences between two reference genomes may actually be polymorphisms rather than fixed divergence. As such, *K*_a _and *K*_s _values calculated from two closely related reference genomes are inflated. This is especially true for *K*_a _because a significant fraction of nonsynonymous polymorphisms can be slightly deleterious mutations destined for elimination before they can reach fixation [11]. This may contribute to the poor quality of *K*_s _in approximating μ and *K*_a_/*K*_s _in approximating selective pressure, and thus the erosion of the positive correlation in short lineages. What remains clear, however, is that polymorphisms are an unlikely source of the positive correlation between ω and *K*_s_. This is because in long lineages, the effect of polymorphisms on *K*_a _and *K*_s _is negligible given that only a tiny fraction of the observed sequence differences between two reference genomes are due to polymorphisms, and yet that a robust positive correlation between ω and *K*_s _can been seen in long lineages. This effectively rules out polymorphisms as a major contributing factor to the correlation.

## Discussion

In this study, we show that the positive correlation between ω and *K*_s _is not restricted to specific mammalian lineages. Rather, the correlation can be observed across all mammalian species for which large-scale genome sequence data are available. It is particularly interesting that the correlation can be observed even in very short lineages once measures are taken to correct for the stochastic noise in *K*_s_. This argues that the failure to observe a positive correlation between ω and *K*_s _in short lineages in our previous study is indeed due to noise in *K*_s _as we had speculated [2]. We note that the calculation of *K*_a _and *K*_s _is minimally confounded by the occurrence of multiple hits in short lineages. The ability to observe a strong positive correlation between ω and *K*_s _in very short lineages therefore argues that the correlation is not an artifact stemming from the improper correction of multiple hits in the calculation of *K*_a _and *K*_s_. Our current study thus bolsters the authenticity of this correlation while demonstrating its broad applicability across the mammalian tree.

Another important message from the study is that *K*_s _in long lineages may provide a much better estimator of neutral mutation rate than *K*_s _in short lineage. This occurs because of the large amount of stochastic variation in *K*_s _relative to the true underlying neutral mutation rate in short lineages than in long lineages. Indeed, when estimating neutral mutation rate of a gene in a short lineage, it may be more accurate to use the observed *K*_s _of that gene in a long lineage (scaled down proportional to the genome-average differential in sequence divergence of the two lineages) than to use the observed *K*_s _from the short lineage. This study and our unpublished data confirm that neutral mutation rate can remain relatively stable across different mammalian lineages [8,9].

Neither the previous long-lineage study nor the current short-lineage study alone is sufficient to eliminate methodological artifacts from consideration. In the study of long lineages, the correlation is complicated by possible difficulties in correcting for multiple hits, though the stochastic noise in *K*_s _and the effect of polymorphisms are not major issues. In the study of short lineages, the converse situation is true. It is thus gratifying that a strong positive correlation between ω and *K*_s _can be observed not only for long lineages, but also for short lineages after noise in *K*_s _is corrected. Together, these results argue that the correlation is the result of a biological mechanism rather than a methodological artifact.

It is as yet unclear why there should exist such a strong positive correlation between ω and *K*_s_. Neither this study nor our previous study provides a definitive mechanism by which this correlation may occur, though several models have been proposed. Perhaps ω is reflective of some combined effect of selective pressure and neutral mutation rate, or perhaps selective pressure and neutral mutation rate influence each other in some way [2]. It was speculated that the occurrence of intragenic compensatory mutations, which is supported by some theoretical and empirical studies [12-14], may contribute to the former scenario [2]. It was also suggested that modulated mutability may contribute to the latter scenario [2], that is, the correlation may be partly due to the fact that genes with conserved functions have evolved lower neutral mutation rates over evolutionary time [15-17]. These speculations notwithstanding, the biological mechanism responsible for the correlation remains unclear.

Given the presence of the correlation in all the mammalian lineages for which there is sufficient genome sequence data, including very short lineages, it now seems clear that the correlation is indeed the result of a biologically meaningful process. The stage is set for future studies to identify the mechanisms underpinning this enigmatic correlation.

## Methods

Using data from Ensembl v36 [18], sequences were obtained for: human, *Homo sapiens *(NCBI 35); chimpanzee, *Pan troglodytes *(PanTro 1.0); rhesus macaque, *Macaca mulatta *(Mmul 1.0); mouse, *Mus musculus *(NCBI m34); rat, *Rattus norvegicus *(RGSC 3.4); and dog, *Canis familiaris *(CanFam 1.0). Genes were clustered into orthologous groups using reciprocal best BLAST hits following established methods [19-21]. We further attempted to ensure proper alignments by imposing maximum acceptable *K*_s _cut-offs at roughly three standard deviations above average for all alignments. Sequences were curated for length differences and for poor alignment. This resulted in 5,831 orthologous groups containing a member from each of the six species with reasonable alignment and 6,779 orthologous groups containing all five primate and rodent species. Ortholog groups, alignments, and evolutionary estimates used in this study can be obtained through the SPEED database [21].

In-frame alignments of orthologs were performed using The Wisconsin Package v10.2 . Evolutionary parameters were estimated using the Li method [22], though the results obtained were comparable (positive linear correlations between ω and *K*_s_) when other methods such as PAML [23,24] were used.

## Competing interests

The author(s) declare that there are no competing interests.

## Authors' contributions

EJV and BTL designed the project, performed analysis and wrote the paper. EJV performed bioinformatic data mining. Both authors have read and approved the final manuscript.

## Supplementary Material

Additional file 1 Supplementary Figure 1. Positive correlation between ω and *K*_s _for the human-mouse lineage and the mouse-rat lineage. Click here for file

Additional file 2 Supplementary Figure 2. Lack of positive correlation between ω and *K*_s _in the human-chimpanzee lineage and the human-macaque lineages. Click here for file

Additional file 3 Supplementary Figure 3. Correlation of *K*_s _or *K*_4 _between different lineages. Click here for file
