# Supplementary material for: Uncovering the mutation-fixation correlation in short lineages
Source: BMC Evol Biol. 2007 Sep 21;7:168. doi: 10.1186/1471-2148-7-168 (PMC2071921; doi:10.1186/1471-2148-7-168)

Supplementary Figure 1. Positive correlation between ω and K_S in the human-mouse lineage and the mouse-rat lineage. The binning of genes follows the convention in Figure 1.

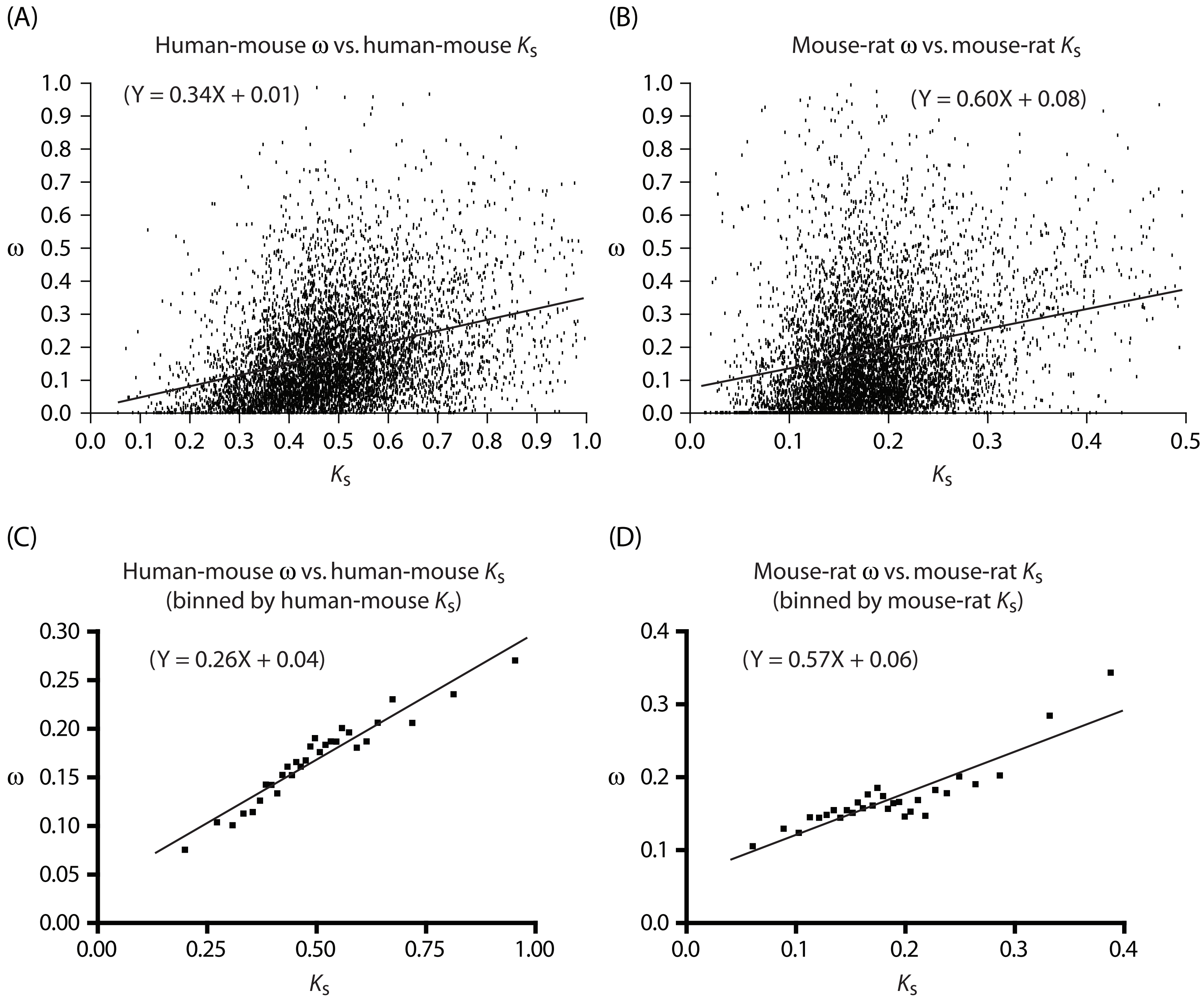

Supplement: Additional file 1 — Supplementary Figure 1. Positive correlation between ω and Ks for the human-mouse lineage and the mouse-rat lineage. [file 1471-2148-7-168-S1.pdf]
