# Supplementary material for: Uncovering the mutation-fixation correlation in short lineages
Source: BMC Evol Biol. 2007 Sep 21;7:168. doi: 10.1186/1471-2148-7-168 (PMC2071921; doi:10.1186/1471-2148-7-168)

Supplementary Figure 2. Lack of positive correlation between ω and K_S in the human-chimpanzee lineage and the human-macaque lineages. The binning of genes follows the convention in Figure 1.

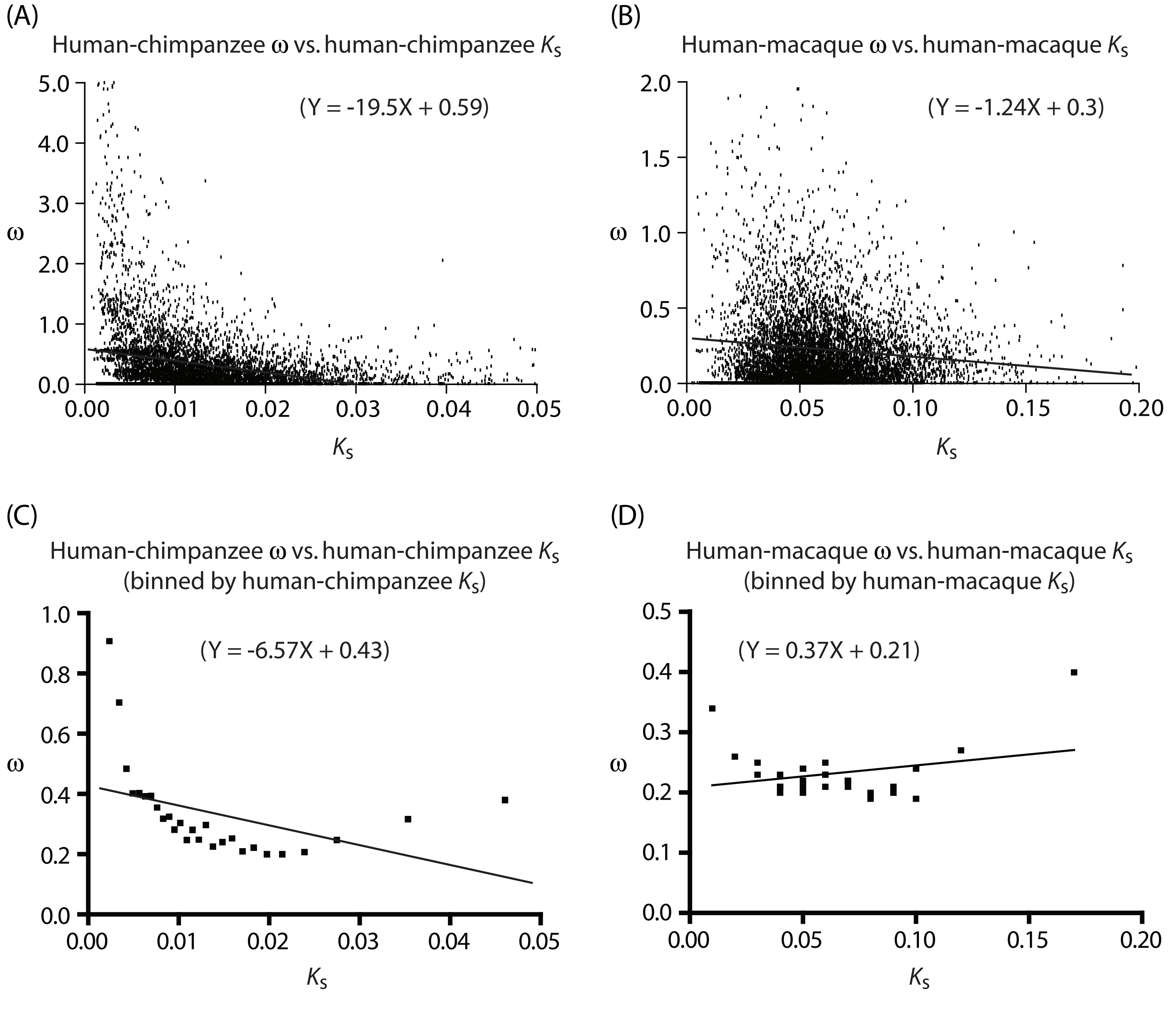

Supplement: Additional file 2 — Supplementary Figure 2. Lack of positive correlation between ω and Ks in the human-chimpanzee lineage and the human-macaque lineages. [file 1471-2148-7-168-S2.pdf]
