# Supplementary figures and images for: Uncovering the mutation-fixation correlation in short lineages
Source: BMC Evol Biol. 2007 Sep 21;7:168. doi: 10.1186/1471-2148-7-168 (PMC2071921; doi:10.1186/1471-2148-7-168)

**Supplementary Figure 3.** Correlation of  $K_S$  or  $K_4$  between different lineages.

(A)

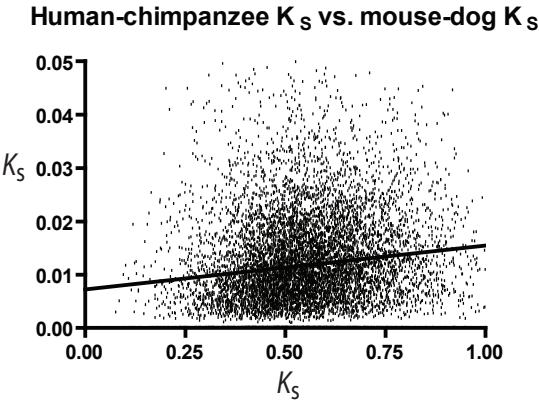

(B)

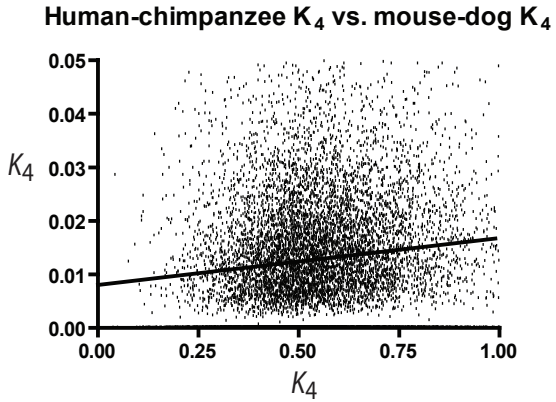

(C)

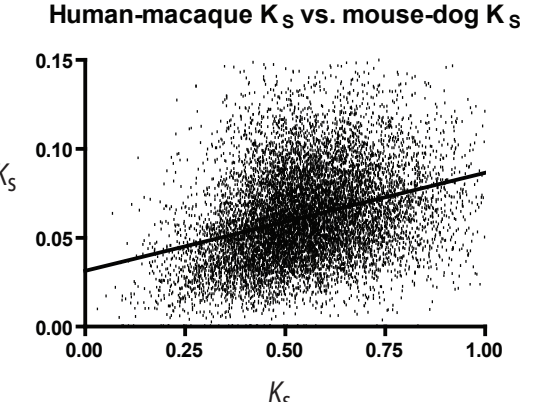

(D)

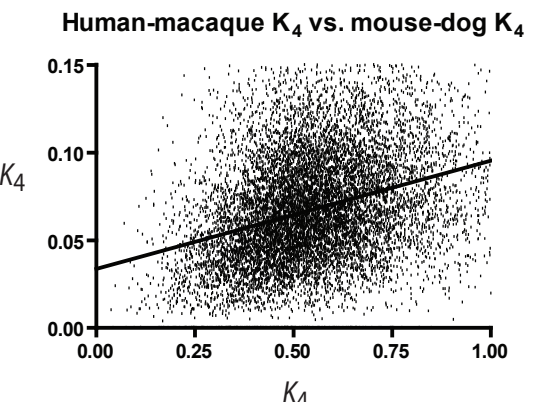

(E)

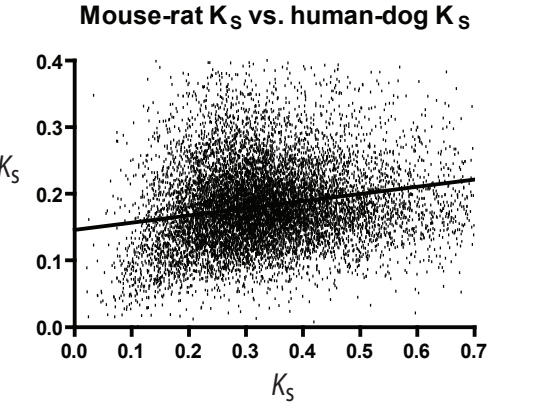

(F)

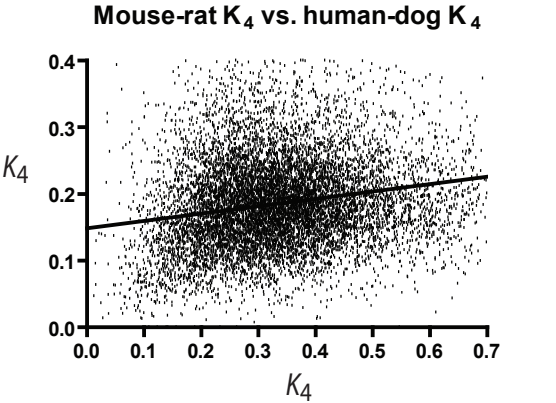

(G)

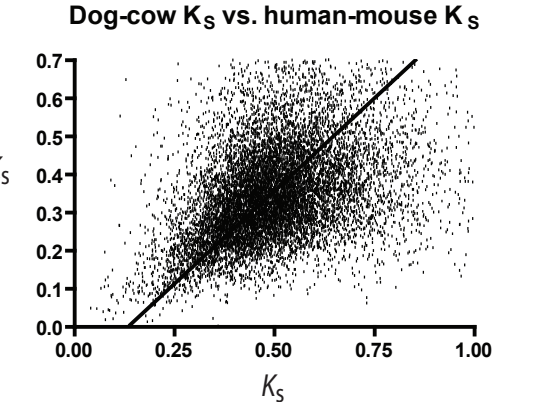

(H)

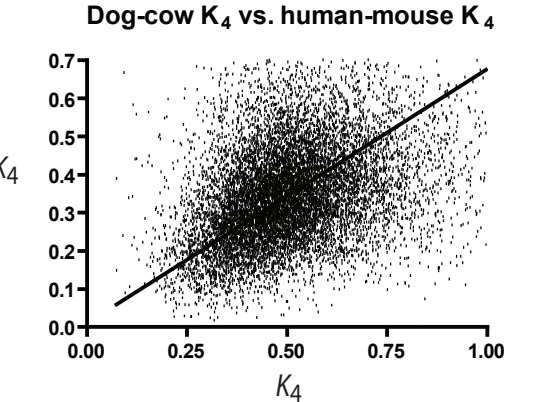

Supplement: Additional file 3 — Supplementary Figure 3. Correlation of Ks or K4 between different lineages. [file 1471-2148-7-168-S3.pdf]
